# Anatomical Connectivity of the Intercalated Cells of the Amygdala

**DOI:** 10.1523/ENEURO.0238-23.2023

**Published:** 2023-10-12

**Authors:** Daniel B. Stern, Anna Wilke, Cory M. Root

**Affiliations:** 1 Department of Neurobiology, School of Biological Sciences, University of California, San Diego, La Jolla, CA 92093; 2 Neuroscience Graduate Program, University of California, San Diego, La Jolla, CA 92093

**Keywords:** amygdala, anatomy, FoxP2, ITC, rabies

## Abstract

The intercalated cells of the amygdala (ITCs) are a fundamental processing structure in the amygdala that remain relatively understudied. They are phylogenetically conserved from insectivores through primates, inhibitory, and project to several of the main processing and output stations of the amygdala and basal forebrain. Through these connections, the ITCs are best known for their role in conditioned fear, where they are required for fear extinction learning and recall. Prior work on ITC connectivity is limited, and thus holistic characterization of their afferent and efferent connectivity in a genetically defined manner is incomplete. The ITCs express the *FoxP2* transcription factor, affording genetic access to these neurons for viral input-output mapping. To fully characterize the anatomic connectivity of the ITCs, we used cre-dependent viral strategies in FoxP2-cre mice to reveal the projections of the main (mITC), caudal (cITC), and lateral (lITC) clusters along with their presynaptic sources of innervation. Broadly, the results confirm many known pathways, reveal previously unknown ones, and demonstrate important novel insights about each nucleus’s unique connectivity profile and relative distributions. We show that the ITCs receive information from a wide range of cortical, subcortical, basal, amygdalar, hippocampal, and thalamic structures, and project broadly to areas of the basal forebrain, hypothalamus, and entire extent of the amygdala. The results provide a comprehensive map of their connectivity and suggest that the ITCs could potentially influence a broad range of behaviors by integrating information from a wide array of sources throughout the brain.

## Significance Statement

The intercalated cells of the amygdala (ITCs) are a fundamental processing structure in the amygdala, yet their anatomy has not been fully characterized. We present a comprehensive input-output mapping of the different clusters using cell-type specific viral approaches combined with quantification of anatomic strength. We confirm the limited existing anatomic data, and importantly, identify previously unknown connectivity with novel implications for function. Moreover, we find that different clusters have unique connectivity, suggesting they serve different processing functions. Thus, we provide for the first time, a full view of the anatomic connectivity of these different ITC clusters. This work should provide a foundation for future studies of this processing center in the amygdala.

## Introduction

The amygdala is a core structure in the brain that is known to be fundamental to a wide range of innate and learned behaviors. In the mouse, the amygdala acts as a processing station for multiple sensory inputs and influences behavior primarily through the central and medial amygdala’s connections to limbic, hypothalamic, and thalamic structures (Janak and Tye, 2015). The intercalated cells of the amygdala (ITCs) are uniquely situated within the amygdala, creating an inhibitory neuronal net surrounding the basolateral amygdala, primarily within the intermediate and external capsules, and extend most of the rostro-caudal length of the amygdala (Millhouse, 1986). They are highly phylogenetically conserved (Moreno and González, 2007; Medina et al., 2011; Yu et al., 2023), GABAergic (Nitecka and Ben‐Ari, 1987; McDonald and Augustine, 1993; Paré and Smith, 1993; Smith and Paré, 1994; Pitkänen and Amaral, 1994; Poulin et al., 2008). They and are often characterized as a ventral extension of the striatum, as they share cytological features, receptor profiles, and a common developmental origin with striatal cells (Kaoru et al., 2010; Busti et al., 2011). Approximately 95% of the ITC cells are medium spiny and project mostly to each other, while the remaining 5% are large, generally aspiny and project mostly outside of the ITC clusters to processing and output nuclei of the amygdala (Millhouse, 1986). However, the full extent of their projections has not been described. They are defined in part by being positive for expressing the *FoxP2* transcription factor, which is essential to their development and maintenance in adult mice (Kuerbitz et al., 2018). The ITCs are behaviorally significant for their role in conditioned fear responses (Likhtik et al., 2008; Hagihara et al., 2021; Rajbhandari et al., 2021). However, even given their known connectivity, the ITCs likely play a role in many amygdala-mediated behavioral responses beyond conditioned fear, and a comprehensive map of their afferent and efferent projections could provide insight into their functions.

Concerted effort to characterize these cells began in the 1980s and continued into the 1990s, with a large emphasis on single and multicell morphologic and electrophysiological characterization. From these studies we learned that ITCs generally project to each other, the central amygdala (CeA), MeA (medial amygdala), BLA (basal lateral amygdala; Mańko et al., 2011) and some parts of the basal forebrain. Each ITC nucleus has varying affinity to these targets that corresponds with distinct receptor profiles. As far as afferents to the ITCs are concerned, much less is known because of the lack of cell type-specific techniques available at the time. The most often described projection to the various ITCs is from the BLA, where the ITCs are described as modulating communication between the BLA and CeA. ITCs have also been shown to receive inputs from hippocampal CA1 pyramidal neurons. They are thought to receive dopaminergic transmission from the ventral tegmental area and serotonin from the dorsal raphe, although it is unclear whether these are direct or volume transmission (Fuxe et al., 2003). There is widespread debate as to whether they receive direct monosynaptic input from the Infralimbic medial prefrontal cortex. The lack of genetic access to ITCs combined with their mesoscopic distribution and topography has precluded a comprehensive and quantitative assessment of their connectome.

To investigate projection targets, we used cre-dependent adeno virus (AAV) vectors in FoxP2-cre mice that label the cytoplasm of infected cells with tdTomato and putative terminals with EGFP conjugated to the synaptophysin protein. To reveal upstream sources of innervation to the ITCs, we used a monosynaptic rabies strategy (Callaway and Luo, 2015). Our results provide a comprehensive map in the inputs and output of the three largest clusters of ITCs, and reveal unique connection profiles for each, particularly for the lateral cluster. These findings have important implications for potential unique functions of the different clusters.

## Materials and Methods

### Virus injections

We used Adult FoxP2-cre male mice (three to nine months old) bred in the animal facility at University of California, San Diego (UCSD) and housed in a reverse 12/12 h light/dark cycle with food and water available *ad libitum*. Animal care protocols and all experiments were approved by the UCSD Institutional Animal Care and Use Committee and were in accordance with the National Institutes of Health guidelines. Transcranial injections of virus were performed on a Kopf stereotactic device model 1900, after mice were deeply anesthetized using isoflurane administered at 2% saturated vapor pressure 1 Lpm, and a local analgesic of Ethica-XR (3.25 mg/kg) or Buprenorphine SR (1 mg/kg) were injected subcutaneously. Mouse blood oxygenation, heart rate and breathing were monitored throughout surgery, and mouse body temperature was regulated using a heating pad (Physio Suite, Kent Scientific). Burr holes were drilled on the dorsal surface of the skull to expose the pial surface of the brain. A glass pipette was lowered into desired injection areas and 20 nL of virus were injected for the synaptophysin virus and the rabies helper virus (Nanoject III, Drummond Scientific). In some cases, the virus was delivered by iontophoresis, whereby 3–5 μA was pulsed for 7 s on and 7 s off for a duration of 2 min. It is noteworthy that iontophoresis provides more localized injections. The data presented contains a mix of the cleanest targeting from both approaches. For the rabies studies, the mice were subsequently injected with EnvA-G-Deleted Rabies-mCherry rabies virus three weeks after injection of the helper virus. The different ITC clusters were targeted by injecting virus at the following stereotaxic coordinates relative to bregma: mITC (AP: −1.06, ML: 2.75, DV: −5.25), cITC (AP: −1.94, ML: 2.5, DV: −5.0), lITC (AP: −1.46, ML: 3.25, DV: −5.0 to −4.5). All viral vectors were purchased from the Salk Institute Gene Transfer, Targeting and Therapeutics Core facility. For anterograde tracing, mice were perfused four to six weeks after surgery. For the rabies studies, mice were perfused 7 d after rabies injections.

### Histology and imaging

Animals were anesthetized by intraperitoneal injection with a mixture of ketamine (100 mg/kg) and xylazine (10 mg/kg). Animals were transcardially perfused first with 10-ml ice-cold 1× PBS and then 10 ml 4% paraformaldehyde (PFA). Brains were extracted and allowed to fix overnight in 4% PFA at 4°C. Brains were sectioned into 75-μm slices using a VT1000 vibratome (Leica) and incubated in primary antibody against eGFP (600-901-B12, Rockland), RFP (600-901-379, Rockland) and FoxP2 (ab1307, Abcam; 1:500 for all primary antibodies) at 4°C overnight. The next day, they were washed three times in 1× PBS for 10 min each wash, then incubated in secondary antibody (1:1000 for all secondary antibodies) overnight again at 4°C. Tissue was then washed three times again in 1× PBS, and then mounted onto glass slides and cover glass using DAPI Fluoromount-G (SouthernBiotech). Imaging of tissue was done exclusively on an Olympus BX61 VS120 Virtual Slide Scanner and 10× objective (Olympus). Exposure settings were held constant across all tissue (DAPI: 80 ms, FITC: 120 ms, TRITC: 120 ms, Cy5: 120 ms).

### Quantification and analysis

Fluorescent images were processed using ImageJ. Olympus .vsi image files were converted into TIFFs using the OlympusViewer plugin, color channels were split, and contrast was enhanced and normalized to account for variance in fluorescence inhomogeneity within and across brain slices. A comprehensive list of target brain areas was first identified by qualitative observation of fluorescence signal, and then all target brain areas were quantified independent of visible fluorescence signal for all ITC inputs or outputs (i.e., if on ITC cluster projected to a given brain area, that area was quantified for all). The abbreviations for relevant brain areas are provided (Table 1). Many brain areas are composed of subnuclei, which were not considered in our analysis. Integrated density was selected in “Set Measurements,” and fluorescence was quantified by using the freeform selection tool and tracing around observable fluorescent area within a given region of interest (ROI), as visually registered to the Franklin and Paxinos atlas (Franklin and Paxinos, 2019). The ROIs delineated in the figures are approximations of general landmarks and not the ROIs used for quantification. For a given ROI, the contralateral hemisphere or adjacent nonexpressing region was measured as a background value, as we did not observe any contralateral projections. Corrected Total Fluorescence was calculated as the Integrated density ROI measurement minus the product of the area of the ROI and mean background fluorescence. This was done for both synaptophysin-eGFP and rabies quantification, as rabies labeling was often too bright and dense to quantify individual cells. The values obtained were also used to construct the Sankey diagram using https://sankeymatic.com/.

**Table 1:** Table of abbreviations

AA	Anterior amygdala nucleus
aCeA	Anterior central amygdala
Aco	Anterior cortical amygdala
AHiAL	Amygdalohippocampal area
aITC	Anterior intercalated cell nucleus
APir	Amygdalopiriform transition area
AUV	Secondary auditory cortex
BLA	Basolateral amygdaloid nucleus, anterior part
BLP	Basolateral amygdaloid nucleus, posterior part
BLV	Basolateral amygdaloid nucleus, ventral part
BMA	Basomedial amygdaloid nucleus, anterior part
BMP	Basomedial amygdaloid nucleus, posterior part
BNST	Bed nucleus of the stria terminalis
CA1py	Hippocampal CA1, pyramidal cell layer
cITC	Caudal intercalated cell nucleus
DLEnt	Dorsolateral entorhinal cortex
DLO	Dorsolateral orbital cortex
EA	Extended amygdala
HDB	Horizontal limb of the diagonal band
InsularCtx	Insular cortex
IPAC	Interstitial nucleus of the posterior limb of the anterior commissure
ITC	Intercalated cells
LA	Lateral amygdaloid nucleus
lITC	Lateral intercalated cell nucleus
MCPO	Magnocellular preoptic nucleus
mdITC	Medial intercalated cell nucleus
MeAD	Medial amygdaloid nucleus, anterodorsal
MeAV	Medial amygdaloid nucleus, anteroventral
MePD	Medial amygdaloid nucleus, posterodorsal
MePV	Medial amygdaloid nucleus, posteroventral
mITC	Main intercalated cell nucleus
pCeA	Posterior central amygdaloid nucleus
PF	Parafascicular thalamic nucleus
PIL	Posterior intralaminar thalamic nucleus
Piri	Piriform cortex
PLCo	Posterolateral cortical amygdaloid area
PMCO	Posteromedial cortical amygdaloid area
PMV	Premammillary nucleus, ventral part
PO	Posterior thalamic nuclear group
PP	Peripeduncular nucleus
PRh	Perirhinal cortex
PSTH	Parasubthalamic nucleus
PVA	Paraventricular thalamic nucleus
RAPir	Rostral amygdalopiriform area
SIB	Substantia innominata, basal part
TeA	Temporal association area
VMH	Ventromedial hypothalamic nucleus
VP	Ventral pallidum
ZIC	Zona incerta, caudal part

## Results

**Figure 1.**
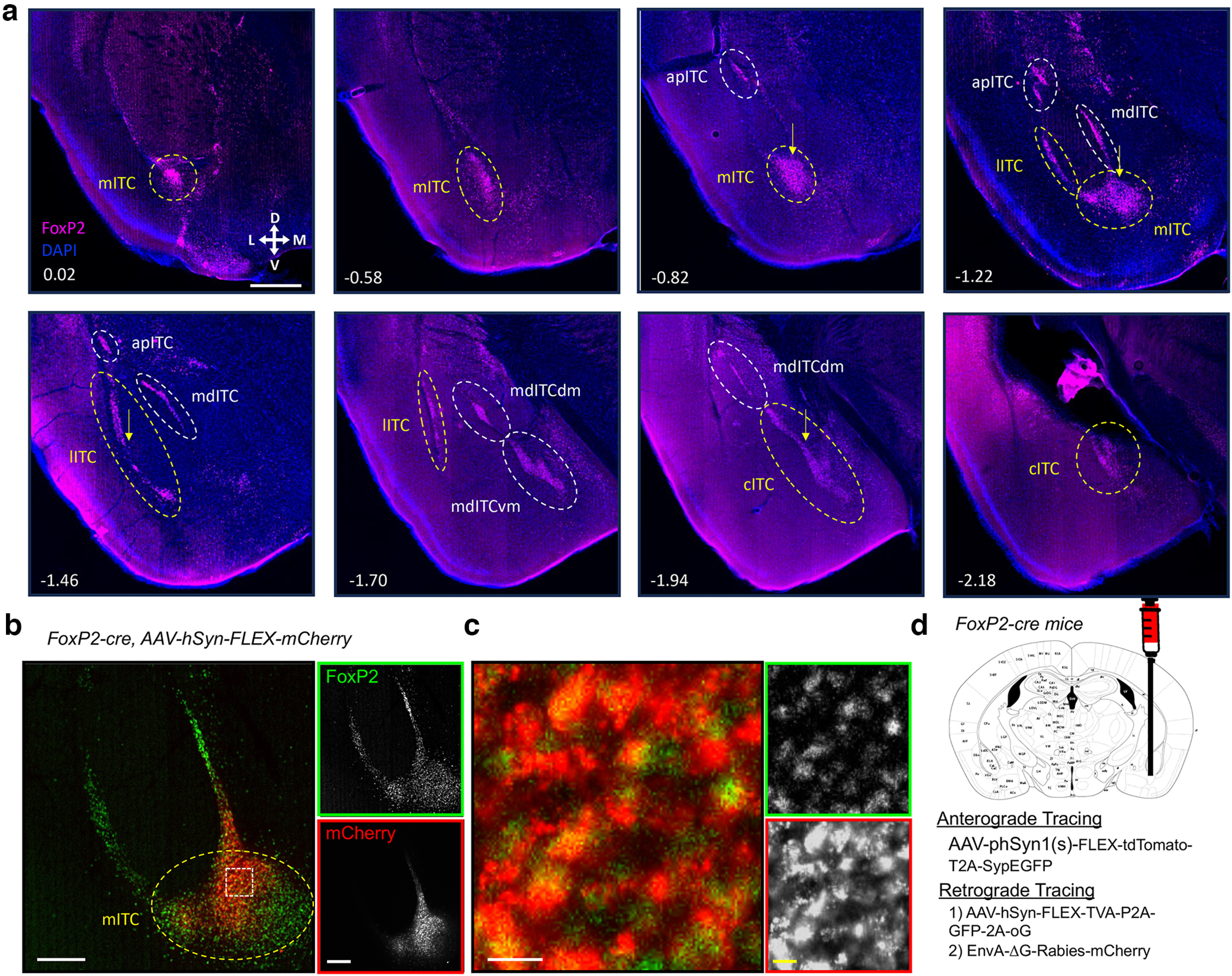
Use of *FoxP2* to target-specific clusters of ITCs. The gene FoxP2 is expressed in ITCs and can be used to define different clusters. ***a***, Immunofluorescence for FoxP2 expression in different regions of the amygdala. Number in lower left corner indicates location of coronal section relative to bregma in the anterior-posterior axis. Scale bar represents ∼500 μm. The clusters are labeled according to prior descriptions. The main (mITC), lateral (lITC), and caudal (cITC) nuclei were targeted for input-output mapping circled in yellow and marked by yellow arrow. ***b***, ***c***, *FoxP2-cre* mice were injected with a cre-dependent mCherry viral vector and tissue was assayed for mCherry and FoxP2 expression. Representative image of the amygdala region where the mITC were targeted with viral vector, see Extended Data Figure 1-1 for targeting other ITC clusters. FoxP2 represented in green, and mCherry in red, in the merged image (left) and individual grayscale images (right). Scale bar is 200 μm. ***c***, Zoomed in image taken from the white box in ***b*** presenting the merged image and individual grayscale channels as in ***b***. Scale bar is 25 μm. ***d***, Schematic of experimental strategy for anterograde and retrograde tracing approach performed in FoxP2-cre mice.

The literature has defined the boundaries of the various ITC nuclei inconsistently because of their unique topology. A recent review (Asede et al., 2022) codified several previous papers (Marowsky et al., 2005; Busti et al., 2011; Hagihara et al., 2021) and offered a codified ITC ontology suggesting 7 distinct definable ITC clusters: apical ITC (apITC), lateral ITC (lITC), anteroventral ITC (aITC), ventromedial ITC (mdITCvm), dorsomedial ITC (mdITCdm), internal ITC (inITC), and posteroventral ITC (cITC). While this is an improved schema, we suggest some additional refinement: (1) we call the aiITC nucleus the main ITC (mITC), a more common term for the cluster; and (2) while they define the medial ITCs (mdITC) as belonging to either ventro-medial or dorso-medial, there is a clear medial ITC band anterior to these more distinct structures that should retain an unqualified medial ITC designation. The mITC is somewhat anatomically distinct from the other ITC clusters as it is more rostral and ventral to the BLA, and not within the intermediate and external capsules. Moreover, it is contiguous with the medial and lateral clusters. FoxP2 expression in these cells is highly similar to previous delineations of this nucleus, suggesting that FoxP2 is a good marker for this nucleus. Thus, we use the traditional main ITC (mITC) terminology, and refer to the pvITC group as the caudal ITC (cITC) nucleus. Importantly, the cITC group is contiguous with the mdITCvm group, and distinguishing them may be somewhat arbitrary. Delineations of the various clusters can be identified by FoxP2 immunostaining (Fig. 1*a*).

10.1523/ENEURO.0238-23.2023.f1-1 Extended Data Figure 1-1 FoxP2-cre line labels FoxP2 ITCs. A cre-dependent reporter (AAV-FLEX-mCherry) was targeted to different ITC clusters in *FoxP2-cre* mice. The brain tissue was stained with a FoxP2 antibody to analyze the overlap in reporter and FoxP2 expression. ***a***, Individual channels and merged images for each of the three ITC clusters. Scale bar is 100 μm. ***b***, Zoomed in image from the white boxes in ***a*** showing the merged image (left) and individual channel (right). Download Figure 1-1, TIF file.

The expression of FoxP2 in ITCs affords a genetic approach to characterizing these neurons. The *FoxP2-cre* mouse line (Rousso et al., 2016) is a targeted *IRES-cre* knock-in behind the endogenous *FoxP2* gene, which should yield cre expression specifically in cells that express *FoxP2*. This line was previously shown faithfully target FoxP2-expressing ITCs (Hagihara, et al., 2021). We confirmed this by injecting a cre-dependent viral vector (AAV-Flex-mCherry) into each of the ITC clusters and assaying for expression of mCherry and FoxP2 by immunoreactivity (Fig. 1*b*,*c*; Extended Data Fig. 1-1). We observed that mCherry-labeled neurons were entirely within the boundaries of the FoxP2 labeling, and close examination indicates that the vast majority of mCherry+ cells were also FoxP2+ for the three ITC clusters examined here. However, we did observe a minority of cells that expressed mCherry but no observable FoxP2, which could result from failure of detection (FoxP2 is nuclear and mCherry cytoplasmic and can reside on different optical planes) or from nonspecific labeling. Nonetheless, the reporter expression appears to largely colocalize with endogenous FoxP2 expression.

### Input-output mapping or ITC clusters

We sought to map the anatomic connectivity of the, main, lateral and caudal ITC clusters using cell-type specific anterorgrade and retrograde tracing in FoxP2-cre mice (Fig. 1*b*). Injections of AAV-phSyn1(s)-FLEX-tdTomato-T2A-SypEGFP-WPRE into FoxP2-cre mice, will selectively express a cytosolic tdTomato and synaptically targeted Synp-eGFP in ITCs, permitting the visualization of synaptic outputs (Wickersham et al., 2007; Wall et al., 2010). We mapped the inputs to these ITC clusters using monosynaptic rabies. The pseudotyped rabies vector EnvA-ΔG- Rabies-mCherry only transfects cells that express the avian TVA protein and it cannot jump across synapses unless the glycoprotein, G, is provided. Thus, injection of AAV-hSyn-FLEX-TVA-P2A-GFP-2A-oG in FoxP2-cre mice will permit subsequent infection of EnvA-ΔG- Rabies-mCherry) to selectively target ITCs and their presynaptic partners. In this scenario, starter ITCs express both GFP and mCherry, whereas presynaptic neurons only express mCherry. Prior work has established that this monosynaptic rabies approach is specific, as elimination of cre eliminates rabies labeling (Wall et al., 2010) and in our experiments elimination of the helper virus prevents rabies expression, and injection of the helper virus in cre-negative mice prevents GFP expression (data not shown).

To quantify the strength of anatomic connectivity, we first qualitatively identified all of the input and output targets to assemble a nearly comprehensive list of brain areas for quantification. In some cases, brain areas, such as the BNST, are composed subnuclei, but we do not distinguish these. However, the projections are visible in the presented images. The total corrected fluorescence was measured for each of the identified brain areas as delineated in the Franklin and Paxinos atlas (Franklin and Paxinos, 2019). This fluorescence signal should scale with density of synaptic outputs, and the number of presynaptic neurons. However, we note that it is not a direct count of synapse or presynaptic neurons, and may be confounded by neurite labeling. Nonetheless, this provides a quantitative assessment of anatomic strength. It is also noteworthy that the total number of inputs to an ITC cluster should scale by the number of starter cells in that cluster. Thus, larger clusters would have more total inputs.

### Main ITC nucleus

**Figure 2.**
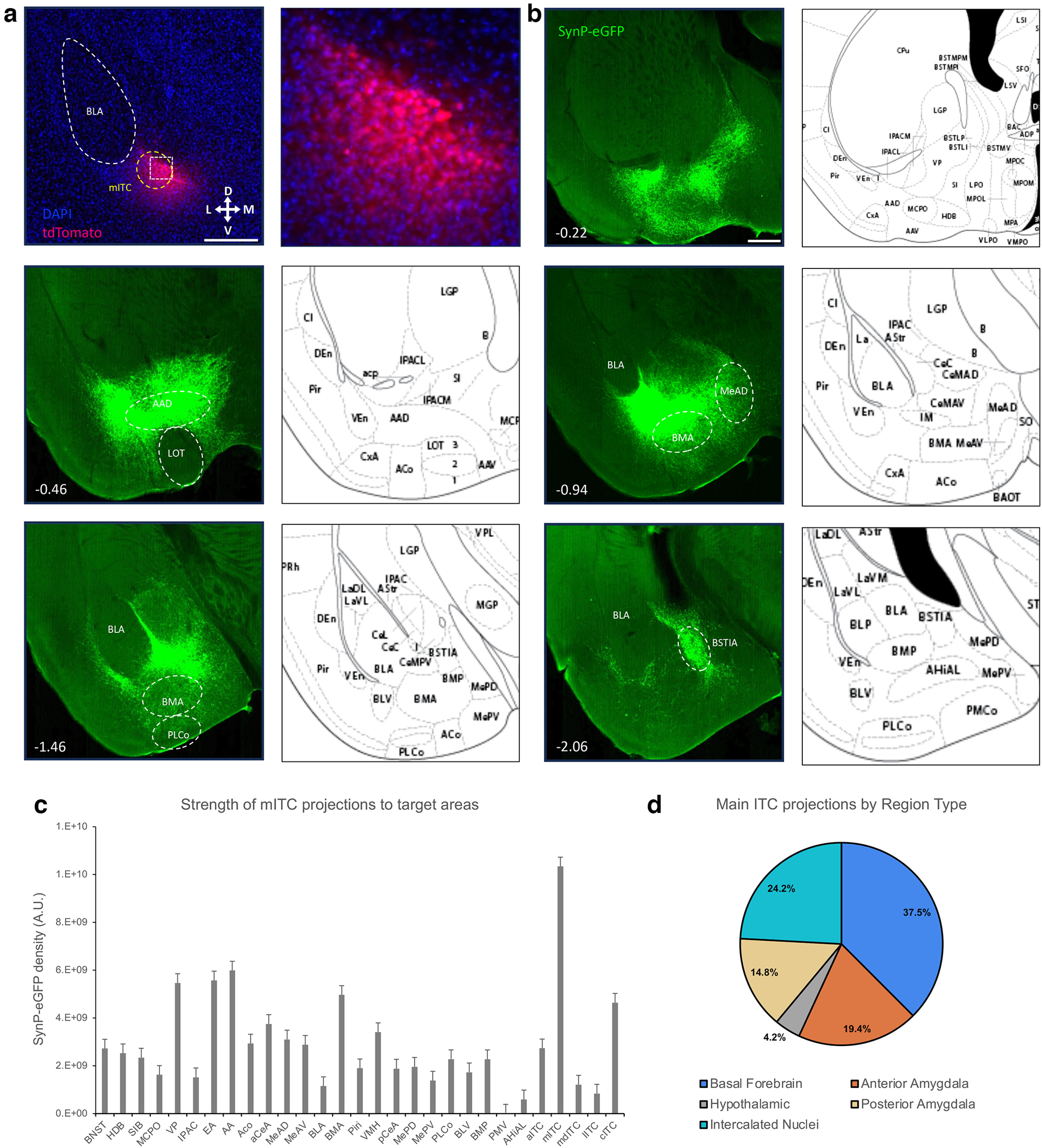
Anterograde tracing of the main ITC cluster. FoxP2-cre Mice were injected with AAV-phSyn1(s)-FLEX-tdTomato-T2A-SypEGFP into the mITC cluster. ***a***, Injection site targeting the main ITCs, showing the cytosolic td-Tomato. Scale bar represents ∼0.5 mm. Number in lower left corner indicates location of coronal section relative to bregma in the anterior-posterior axis. The white box depicts a region shown at higher magnification (right), image is ∼200 × 200 μm. ***b***, Representative images of projection targets identified by synaptophysin-eGFP expression. ***c***, Corrected Total Fluorescence data for regions targeted by the mITC (*n* = 3). Error bars show SEM. ***d***, Pie charts depicting the category of regions targeted by the mITC cluster.

We first sought to define the synaptic targets of the mITC neurons by targeting Injections of AAV-phSyn1(s)-FLEX-tdTomato-T2A-SypEGFP-WPRE into the mITC cluster in FoxP2-cre mice (Fig. 2*a*). Targeting to the mITC nucleus revealed broad and dense projections to other ITC nuclei, significant portions of the amygdala, and especially the basal forebrain (Fig. 2*b–e*), and all projections were ipsilateral. Specifically, within the amygdala, we observed dense projections to the entirety of the AP extent of the medial amygdala, significant projections to most portions of the cortical amygdala and basomedial amygdala, and consistent projections to the central amygdala throughout the AP axes. Surprisingly, we observed very little evidence of projections into the basolateral amygdala, as previously suggested (Marowsky et al., 2005; Mańko et al., 2011; Asede et al., 2022; Strobel et al., 2015). Within the basal forebrain, we observed dense projections to the horizontal limb of the diagonal band, MCPO, substantia innominata, and ventral pallidum. Interestingly, although the BNST and basal forebrain regions are adjacent, projections from the mITC to these regions appear to take separate routes. Projections to the BNST appear to go caudally through the cITCs, through the stria terminalis and enter the BNST from the dorsal aspect. Although the BNST is composed many subnuclei, for simplicity, we do not distinguish them here, but they are visible in the representive images. Projections to the basal forebrain from the mITCs go directly rostral from the mITC and innervate the vast majority of the basal forebrain, while having a clear border along the dorsal tubercle and lateral hypothalamic areas.

**Figure 3.**
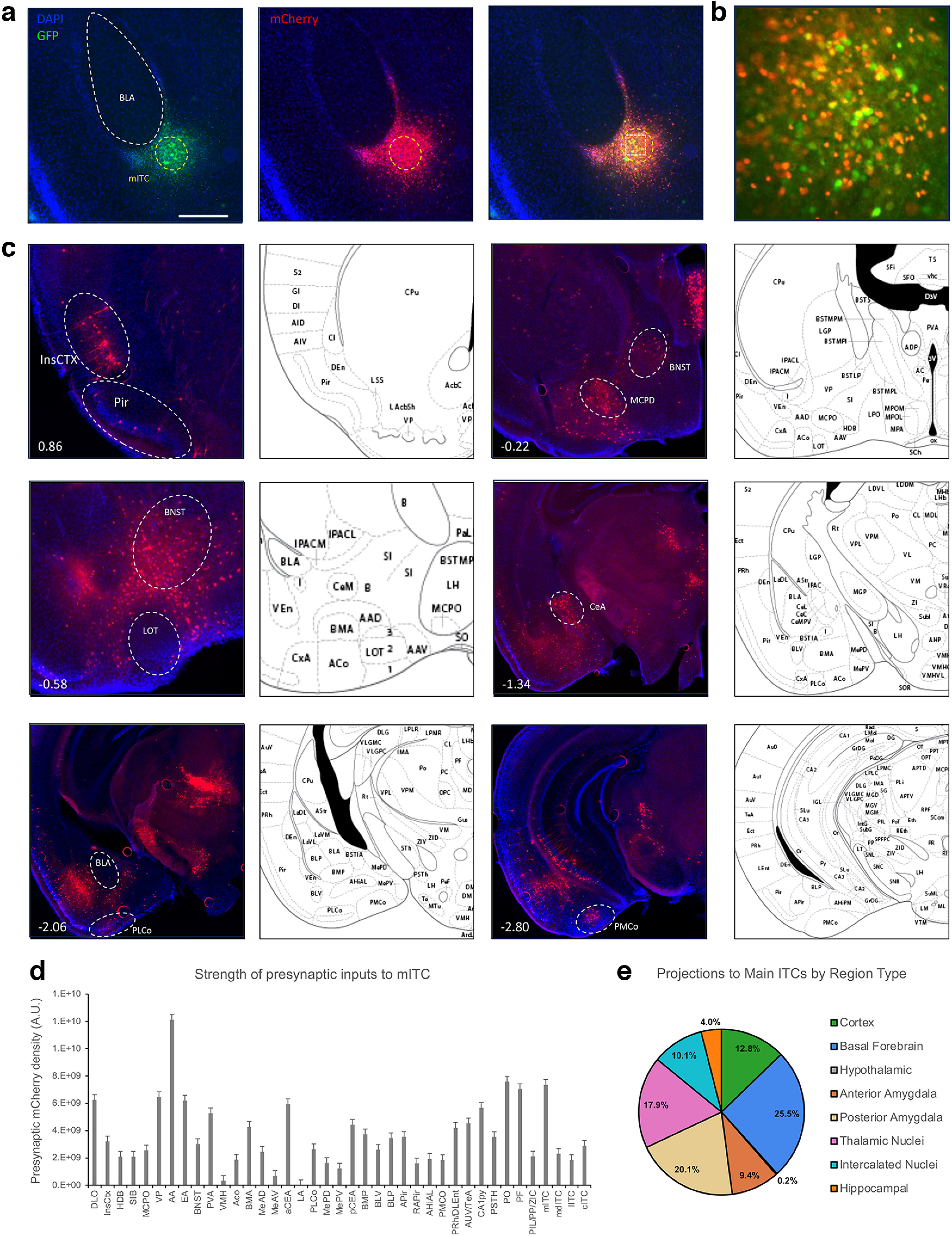
Retrograde tracing of inputs to the main ITC cluster. Monosynaptic rabies vectors, AAV-hSyn-FLEX-TVA-P2A-GFP-2A-oG and EnvA-G-Deleted Rabies-mCherry, were injected into FoxP2-cre mice to identify the inputs to the mITC cluster. ***a***, Representative coronal section showing targeting of the helper virus (green) and rabies (red) in the mITCs. Scale bar represents ∼0.5 mm. White box in merged image outlines the region shown in ***b*** of starter cells in the mITC cluster, image is ∼200 × 200 μm. ***c***, Representative images show regions identified to have presynaptic neurons identified by mCherry expression. Number in lower left corner indicates location of coronal section relative to bregma in the anterior-posterior axis. ***d***, bar graphs show corrected total fluorescence quantification of the presynaptic regions (*n* = 3). Error bars show SEM. ***e***, The pie chart depicts the category of regions that project to the mITC cluster.

Next, we sought to map the inputs to the mITC nucleus using monosynaptic rabies in FoxP2-cre mice (Fig. 3*a*,*b*). Inputs to the mITC nucleus as revealed by mCherry expressing presynaptic neurons (Fig. 3*c*) reveal that the mITC is reciprocally connected with many of its target areas, while receiving additional input from several thalamic structures and cortical areas (Fig. 3*d*,*e*). The densest projections to the mITC originate in the ethmoid and posterior thalamic nuclei, paraventricular thalamic nucleus, and pyramidal layer of hippocampal CA1 region. However, it also receives substantial projections from various amygdalar nuclei including the anterior amygdala (AA), medial amygdalae, central amygdalae, posterolateral cortical amygdala, posteromedial cortical amygdala, and amygdalapiriform transition area. We observe scattered but abundant numbers of presynaptic neurons in the basal forebrain especially the ventral pallidum, substantia innominata, horizontal limb of the diagonal band, and medial forebrain bundle MCPO. Additionally, we consistently observed rabies-labeled neurons in the insular cortices along most of their entire AP extent, the entorhinal cortices, as well as the AUV/TeA. Surprisingly, no presynaptic neurons were observed in the Infralimbic or prelimbic cortices, lending support to the existing evidence suggesting they are not monosynaptically connected to the ITCs (Strobel, et al., 2015), despite some electrophysiological evidence to the contrary (Cho et al., 2013).

### Caudal ITCs

**Figure 4.**
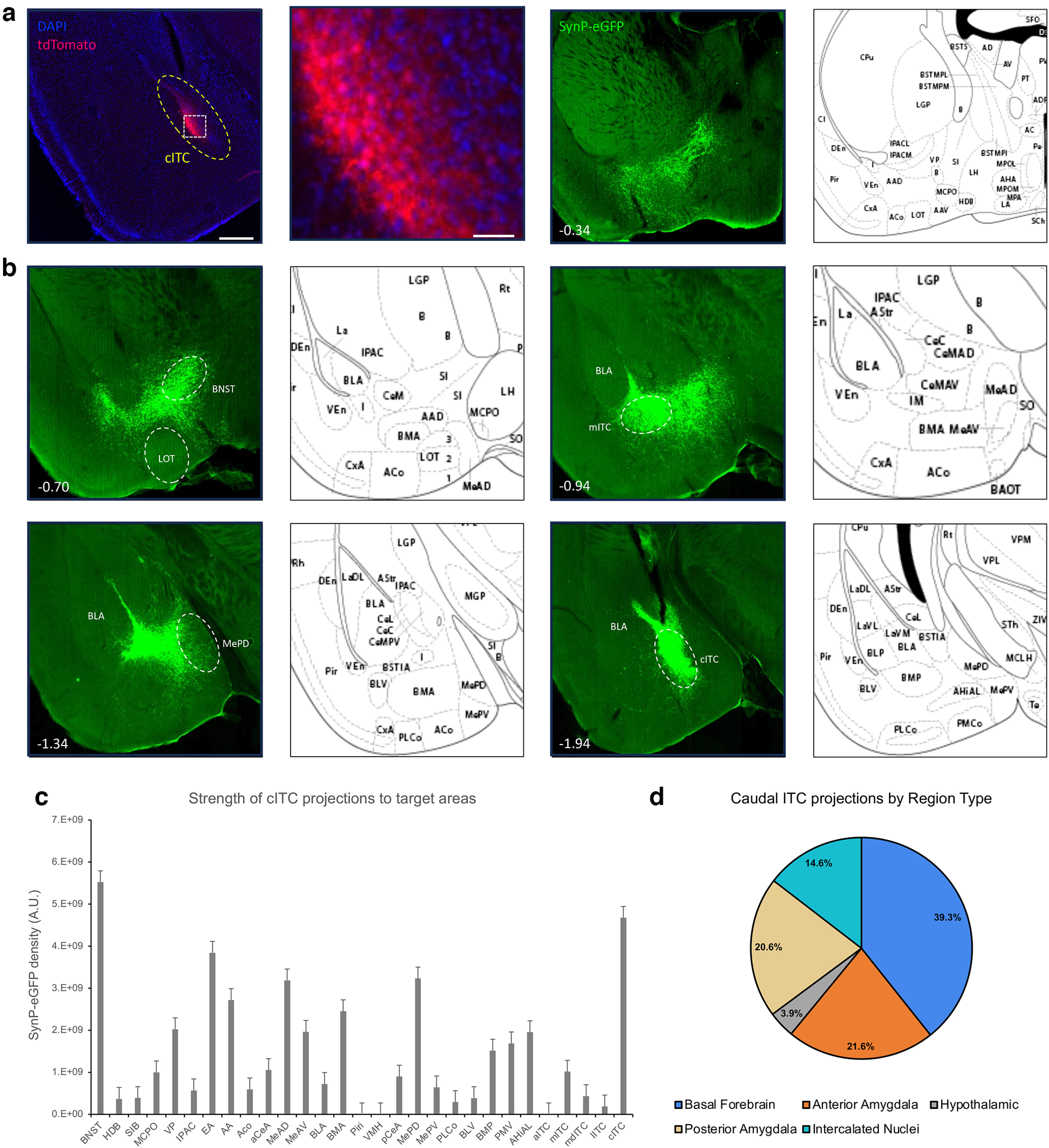
Anterograde tracing of the caudal ITC cluster. FoxP2-cre Mice were injected with AAV-phSyn1(s)-FLEX-tdTomato-T2A-SypEGFP into the cITC cluster. ***a***, Injection site targeting the cITCs, showing the cytosolic td-Tomato. Scale bar represents ∼0.5 mm. The white box depicts a region shown at higher magnification (right), scale bar is ∼50 μm. ***b***, Representative images of projection targets identified by expression of synaptophysin-eGFP. Number in lower left corner indicates location of coronal section relative to bregma in the anterior-posterior axis. ***c***, Corrected total fluorescence data for regions targeted by the cITC (*n* = 3). Error bars show SEM. ***d***, Pie charts depicting the category of regions targeted by the cITC cluster.

Anterograde tracing of the cITCs (Fig. 4) reveals that this nucleus largely projects to a similar set of the regions targeted by the mITC nucleus, with some minor differences. Generally, they show similar profiles in their broad and dense projections to other ITC nuclei, significant portions of the amygdala, and the basal forebrain, and all projections were ipsilateral. However, some important differences do exist. Anteriorly, the cITCs project less anteriorly than the mITC nucleus. Dense axon fields are detected within the more caudal basal forebrain including the anterior amygdala (AA) and extended amygdala (EA) regions. Similar to the mITC nucleus, the nucleus of the lateral olfactory tract (nLOT) is completely spared, but unlike the mITC nucleus we observe very few axons in any of the CeA. The densest projections appear to be within the MeA, particularly the anterior and caudal dorsal MeA nuclei, and the mITC nucleus itself. While we do observe some fluorescence in the BMA and CoA, these projections are comparatively light (Fig. 4).

**Figure 5.**
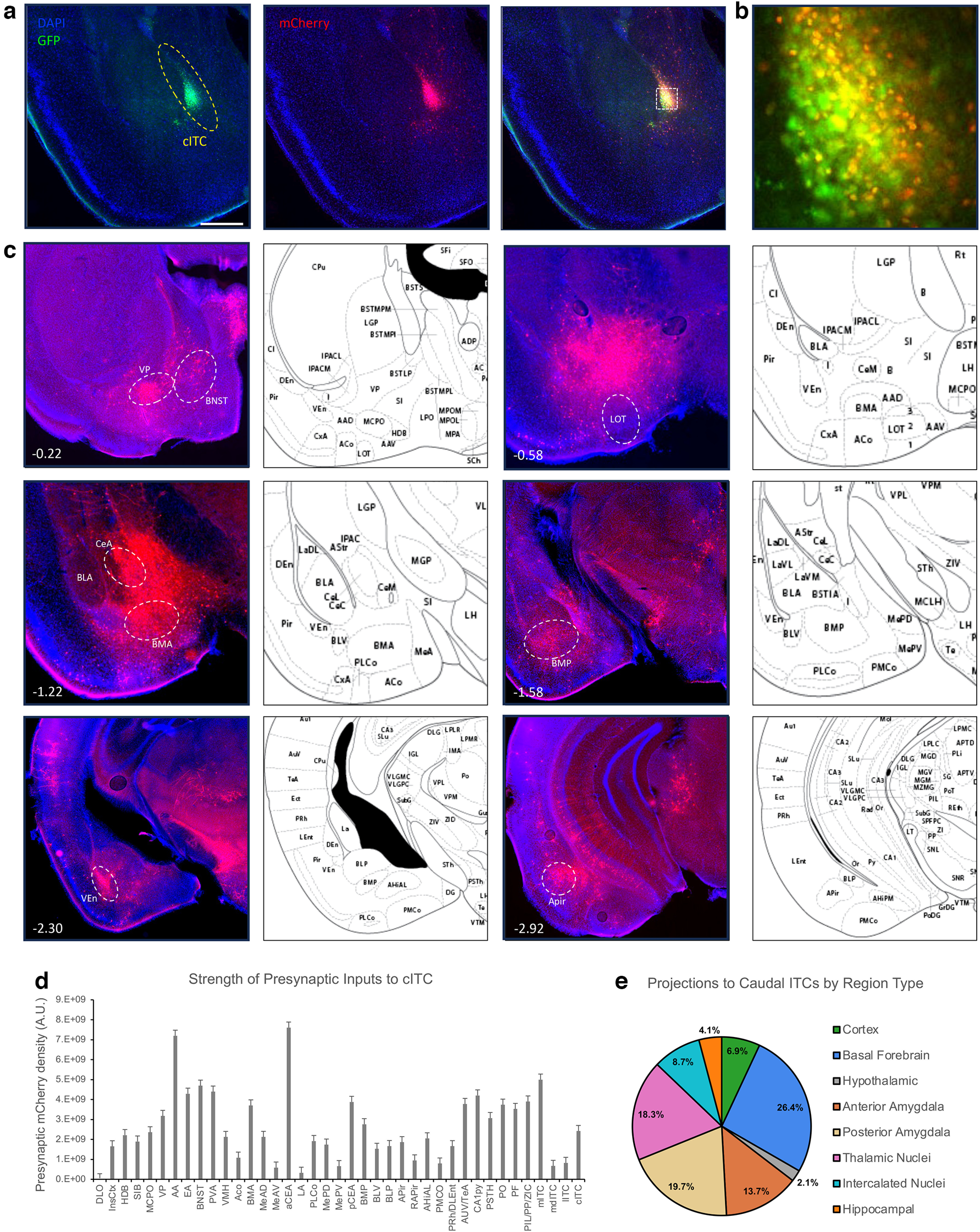
Retrograde tracing of inputs to the caudal ITC cluster. Monosynaptic rabies vectors were used to trace the inputs to the cITC cluster. ***a***, Representative coronal section showing targeting of the helper virus (green) and rabies (red) in the cITCs. Scale bar represents ∼0.5 mm. White box in merged image outlines the region shown in ***b*** of starter cells in the mITC cluster, image is ∼200 × 200 μm. ***c***, Representative images show regions identified to have presynaptic neurons identified by mCherry expression. Number in lower left corner indicates location of coronal section relative to bregma in the anterior-posterior axis. ***d***, bar graphs show corrected total fluorescence quantification of the presynaptic regions (*n* = 3). Error bars show SEM. ***e***, The pie charts depict the category of regions that project to the cITC cluster.

Monosynaptic rabies tracing reveals that the cITCs (Fig. 5) receive information from a similar set of distributed brain regions as the mITCs. Anteriorly, we observe clusters within the Insular cortices. The basal forebrain nuclei have many presynaptic neurons, and we also observe dense presynaptic clusters within the PVA. The BNST similarly has moderately dense presynaptic clusters. cITCs receive information from all the same amygdala regions as the mITC, and in similar proportion across regions. Similar to the mITC nucleus, the cITCs also receive projections from dense clusters within posterior regions: cortices including the Prh, DLEnt, AUV, and TeA; the ventral hippocampal CA1 pyramidal cell layer; and especially thalamic nuclei including the PSTH, PO, PF, PIL, PP, and ZIC. Given the overlap in both projection targets and sources of innervation between cITCs and mITCs, it will be important to discover whether the cellular identities in these regions are the same or different, and to what degree they overlap.

### Lateral ITCs

**Figure 6.**
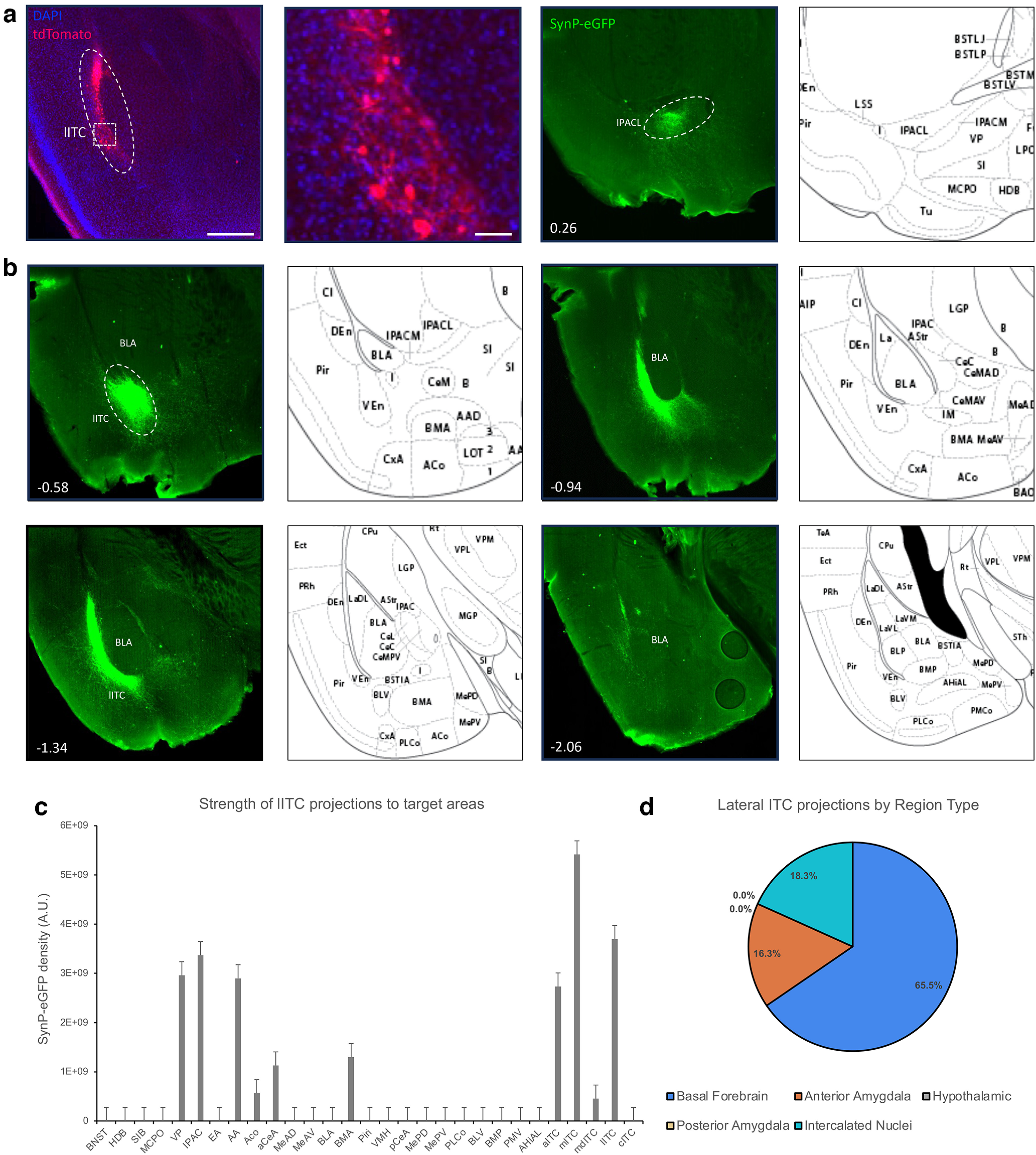
Anterograde tracing of the lateral ITC cluster. FoxP2-cre mice were injected with AAV-phSyn1(s)-FLEX-tdTomato-T2A-SypEGFP into the lITC cluster. ***a***, Injection site targeting the lITCs, showing the cytosolic td-Tomato. Scale bar represents ∼0.5 mm. The white box depicts a region shown at higher magnification (right), scale bar is ∼50 μm. ***b***, Representative images of projection targets identified by synaptophysin-eGFP expression. Number in lower left corner indicates location of coronal section relative to bregma in the anterior-posterior axis. ***c***, Corrected total fluorescence data for regions targeted by the lITC (*n* = 3). Error bars show SEM. ***d***, Pie charts depicting the category of regions targeted by the lITC cluster.

Based on afferent and efferent connectivity, the lateral ITCs stand out among the nuclei studied here. Anterograde tracing (Fig. 6) reveals that they project almost exclusively to the other ITC nuclei, and barely touch either the amygdala output nuclei or the basal forebrain, and all projections were ipsilateral. Their primary projection is to the mITC nucleus, with a dense projection reaching the anterior ITC nucleus and some axons extending up into the medial ITC cluster. We also see a very light projection into the BMA, aMeA, plCoA, and even some axons that project toward the piriform cortex (not quantified). These data suggest that the primary role of the lITCs work mostly to modulate the activity of the other ITC nuclei, with minimal direct impact on the primary processing and output nuclei of the amygdala and the basal forebrain.

**Figure 7.**
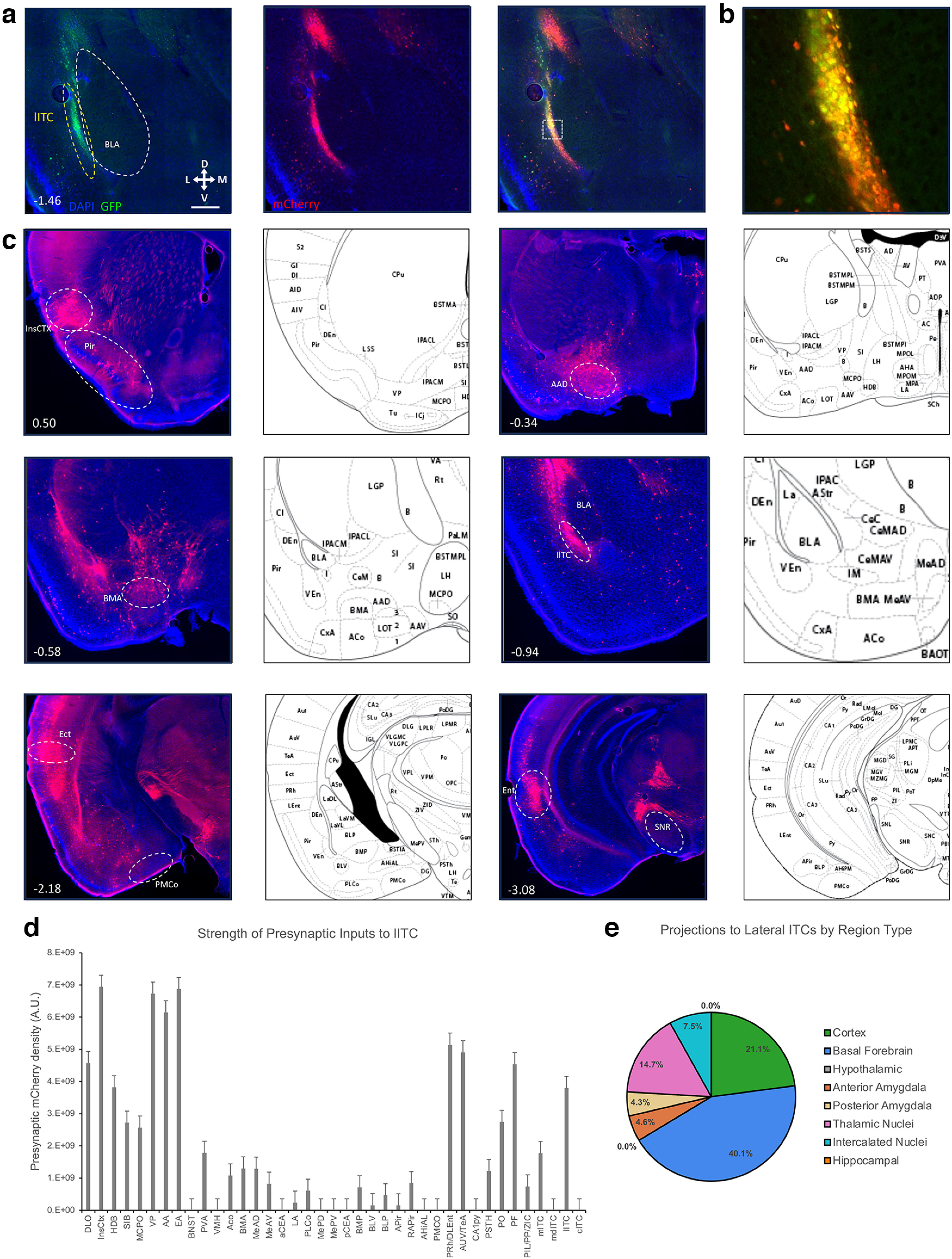
Retrograde tracing of inputs to the lateral ITC cluster. Monosynaptic rabies vectors were used to trace the inputs to the lITC cluster. ***a***, Representative coronal sections showing targeting of the helper virus (green) and rabies (red) in the lITCs. Scale bar represents ∼0.5 mm. White box in merged image outlines the region shown in ***b*** of starter cells in the mITC cluster, image is ∼200 × 200 μm. ***c***, Representative images show regions identified to have presynaptic neurons identified by mCherry expression. Number in lower left corner indicates location of coronal section relative to bregma in the anterior-posterior axis. ***d***, bar graphs show corrected total fluorescence quantification of the presynaptic regions (*n* = 3). Error bars show SEM. ***e***, The pie charts depict the category of regions that project to the lITC cluster.

Monosynaptic rabies also reveals that the lITCs (Fig. 7) have a unique connectivity profile from the other ITC clusters. Anteriorly, the lITCs receive projections from the lateral OFC. This group of presynaptic neurons extends continuously into the insular cortices for the majority of their AP length. While the lITCs also receive projections from the PVA, this source of innervation is considerably less than those received by the mITC and cITC nuclei. Like the other ITC nuclei, the lITCs receive robust input from the basal forebrain regions. Unlike the other ITC nuclei, lITCs receive very few projections from the processing and output stations of the amygdala, including the BLA, BMA, CoA, and MeA, with the most presynaptic neurons evident in the MeA, BMP, and a few in the BLA. Caudally, the lITCs differentiate themselves with a significant projection from midline thalamic nuclei including the interomediodorsal thalamic nucleus (IMD), posteromedian thalamic nucleus (PoMn), and mediodorsal thalamic nucleus (MDM). Additionally, dense clusters of presynaptic neurons were observed in the shell of the reticular substantia nigra (SNR), posterior thalamic nucleus (PoT), and the dorsolateral entorhinal cortex (DLEnT).

**Figure 8.**
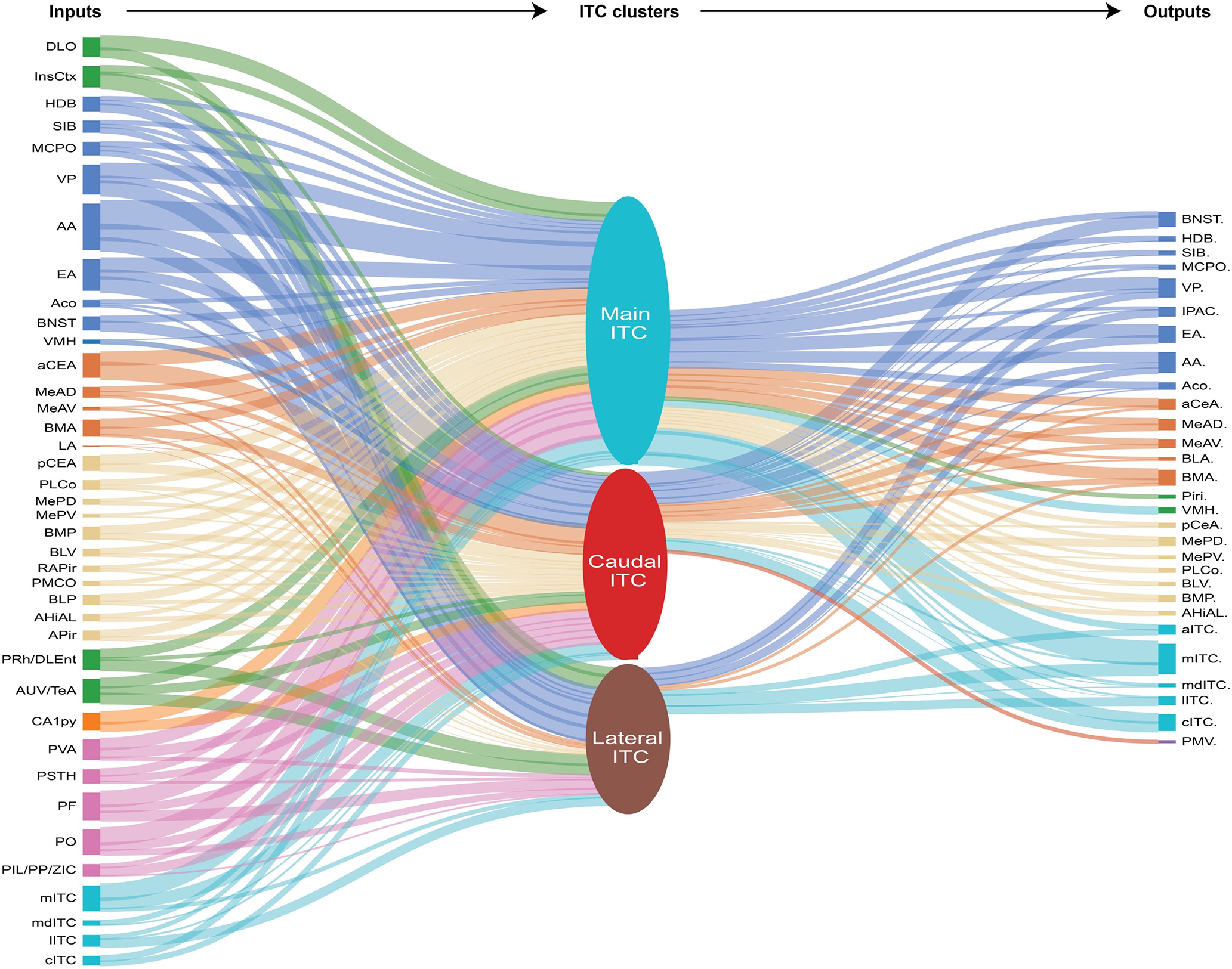
Schematic representation of the relative strength of afferent and efferent connections with the three ITC clusters. A summary diagram representing the inputs and outputs of the different ITC clusters. Line thickness represents the relative anatomic connection strength, measured by fluorescence signal, between presynaptic inputs (left column of regions) and projection targets (right hand column of regions) of the three ITC nuclei evaluated.

Lastly, we sought to quantify the inputs and outputs of each ITC cluster to allow for comparison between the groups using a visual diagram. We present a summary schematic (Fig. 8) identifying the relative anatomic strength of each ITC cluster’s inputs and outputs. Thus, these findings provide a comprehensive view of the ITC connectivity for these prominent clusters.

## Discussion

Holistic characterization of the ITCs connectivity affords a foundation for understanding the role of this phylogenetically conserved inhibitory system within the amygdala. Until now, this level of characterization has remained elusive largely because of the lack of specific genetic access and modern systems level neuroscience tools. While previous studies have demonstrated some connectivity between many of the regions discussed here, projections outside of the amygdala had mostly not been identified and the relative density of these connections have thus far remained unquantified. Thus, our results reveal novel projections from the ITCs to regions outside the amygdala. The use of FoxP2-cre for specific targeting provides an approach for comprehensive anatomic mapping and will be useful for cell-type specific manipulations. Our work suggests that these distributed nuclei could play a fundamental role in amygdala function, well beyond what we currently know about their involvement in conditioned fear. Our presynaptic rabies tracing demonstrates that they receive input from distributed brain systems that perform high-level and low-level processing, suggesting that the ITCs are integrators of disparate information. Our anterograde tracing demonstrates that they preferentially and densely innervate the primary processing and output stations of the amygdala as well as key regions of the basal forebrain. This suggests they potentially act as either an inhibitory check or modulator of limbic processing and decision-making before action implementation and physiological adjustment, as well as inhibitory control of cholinergic innervation to the cortical mantle. Additionally, the extensive interconnectivity between the ITC nuclei combined with their distribution of medium spiny and large aspiny neurons suggests they perform complex computations like the basal ganglia that will be parsed in future studies.

Important findings in this work include what was not observed. For instance, projections from the BLA to the ITCs is a commonly discussed synapse in ITC field (Palomares-Castillo et al., 2012; Asede et al., 2022); however, our rabies tracing showed very little input from the BLA to ITCs, indicating that this connection reflects a minor subpopulation and that the ITCs likely serve broader functions than currently known or suggested. Instead, based on density of presynaptic regions, ITCs are more likely driven by cortical and thalamic nuclei that may present an unremarked confound in BLA-ITC studies. Alternatively, studies on conditioned fear looked at mdITC connections, which was not under direct investigation here, and it may be that the BLA projects principally to this ITC nucleus (Hagihara et al., 2021). We found a similar lack of evidence for a strong connection with the LA, which may be explained by a similar rationale. Lastly, several papers have described a direct monosynaptic connection between the infralimbic cortex and the ITCs which purportedly explains prefrontal control over conditioned fear responses (Cho et al., 2013). We find no evidence of a direct monosynaptic connection between these regions, but instead observe some evidence of possible prefrontal control of the mITC stemming from the lateral OFC.

The FoxP2-cre mouse is an important advancement in our ability to study this enigmatic structure, however it is worth noting some limitations on its use. The FoxP2 transcription factor has been demonstrated to be key to ITC cell maintenance and survival. However, there exists within the amygdala, basal forebrain, and striatum many FoxP2+ cells that do not reside strictly within the classically delineated as ITC nuclei. This confound is particularly noticeable in the Mea, CoA, and BMA where ample FoxP2+ cells are found. This, coupled with the close proximity of the various ITC nuclei, makes surgically targeting these nuclei with precision extremely difficult, enhancing the likelihood of observing off-target effects in both anatomic and behavioral experiments. FoxP2+ cells in the striatum are problematic if backflow is observed. Here, we have chosen the best examples of selective targeting we were able to achieve. Iontophoretic injection of virus proved to be invaluable in selective targeting of these nuclei relative to pressure injection, although examples of both are presented here.

ITCs in the nuclei around the amygdala are characterized by the expression of FoxP2, however FoxP2 is expressed outside of these boundaries. These cells likely share developmental origins but may serve entirely different functions. An alternative interpretation of FoxP2+ positive cells found outside typical ITC nuclei boundaries is that they should be characterized as “extended” ITC cells, particularly those that appear to emanate from the medial boundary of the mITC nucleus. However, genetic sequencing and additional developmental studies will need to be conducted to evaluate this ontological hypothesis. Looking at FoxP2 distribution throughout the basal forebrain also suggests the possibility that the ITCs are but one instance of an inhibitory computational schema that extends rostrally well beyond the amygdala. The mesoscopic defining characteristic of ITCs is that they are densely bound clusters within and along boundaries between adjacent brain regions. A cursory examination of FoxP2+ cell distribution suggests that this motif is repeated throughout the ventral forebrain, with many FoxP2+ dense clusters observed within the ventral pallidum, olfactory tubercle where the islands of Calleja are densely FoxP2+, within and surrounding the nucleus accumbens, delineating the boundary between the piriform and striatum/ventral pallidum, and all the way to the tenia tecta where dense FoxP2+ clusters separate them from the accessory olfactory areas. Future work should examine whether these dense FoxP2+ clusters share similar developmental origin, genetic and receptor expression profiles, connectivity, and functional homology to the classic ITCs.

In summary, our work for the first time demonstrates the relative anatomic strength of connectivity between afferent and efferent projections to and from the three largest and separable ITC nuclei. This quantitative assessment suggests potential discrete functionality between these nuclei, and a rational platform for novel hypotheses. Thus, this anatomic framework affords a foundation for the future dissection of ITC circuit physiology and function.

## Synthesis

Reviewing Editor: Sam Golden, The University of Washington Decisions are customarily a result of the Reviewing Editor and the peer reviewers coming together and discussing their recommendations until a consensus is reached. When revisions are invited, a fact-based synthesis statement explaining their decision and outlining what is needed to prepare a revision will be listed below. The following reviewer(s) agreed to reveal their identity: Kurt Fraser.

This manuscript was reviewed by two experts in the field. Both reviewers felt that the manuscript provided valuable information for the field in relation to ITC efferent and afferent connectivity, as defined by Fox2p expression, and that this is an interesting and important topic due to ITC’s known roles in the regulation of many behaviors. It was specifically noted that this is an exciting research direction of an underappreciated aspect of heterogeneity in the amygdala.

However, the following methodological concerns were noted by reviewers, centered on either the use of Fox2P as a marker, how ROI quantification was performed, and validation of rabies tracing:

Fox2p:

1) The expression of the viral constructs appears to occupy a much larger area than the authors identify with the FOXP2 cell line (see expression of tdtomato in Fig 2A and 6A). I note this because the images of expression are from immunofluorescence of the FOXP2 protein and not from a labeled genetic cross or expression within the mouse line used in all other work. Did the authors confirm the specificity of viral labeling of tdtomato with FOXP2? I appreciate the zoomed out images, but even the zoomed in snapshots are difficult to see or support that the labeling is to cell bodies.

2) The authors refer to the mITC as a cluster based on foxp2 expression. This seems quite anatomically distinct from the other groups, almost as if it is simply part of the basalmedial amygdala vs. intercalated cells. This is discussed in the manuscript, but I am not sure simply using the marker of foxp2 is enough to keep calling them mITC. I do note, this is my opinion, and the authors discussion is sufficient, I just wanted to note.

ROI quantification:

3) The authors suggest the ‘BNST’ as a key output of several of these structures. There needs to be more precision here. For example in figure 2, the top left panel, there is clear outputs to the BSTLP. but it is not marked or delineated. Whereas in the lower panel, the BSTIA is delineated as BNST. More precise subregion descriptors is required.

4) In this vein, the ROI used for quantification seems a bit imprecise based on the figures. It may be better to align images based on the actual atlas. In addition, the use of the contralateral hemisphere as background would only be appropriate if there was no projection, is this the case?

Rabies tracing:

5) There is no control for the rabies tracing. The authors do not quantify starter cells or normalize input strength to starter cells. There is known leakiness in rabies tracing and there is a need for control experiments injecting either viral construct separately and quantifying upward inputs. As is, there is no ability to assess whether the inputs are indeed faithful to the inferred mechanism of action of this approach.

## Author Response

We thank the reviewers for the helpful comments. We have made edits to the manuscript for each of the reviewer’s points and respond below. Modified text in the manuscript is highlighted in yellow.

Fox2p:

1) The expression of the viral constructs appears to occupy a much larger area than the authors identify with the FOXP2 cell line (see expression of tdtomato in Fig 2A and 6A). I note this because the images of expression are from immunofluorescence of the FOXP2 protein and not from a labeled genetic cross or expression within the mouse line used in all other work. Did the authors confirm the specificity of viral labeling of tdtomato with FOXP2? I appreciate the zoomed out images, but even the zoomed in snapshots are difficult to see or support that the labeling is to cell bodies.

Response: This is indeed an important point. The expression of the viral constructs is entirely within the boundaries of the FoxP2 delineated nuclei. The area may have appeared larger due to the display settings of the zoomed out images, which we have replaced. Further, we have added evidence to validate the Foxp2-cre line as a specific marker for these neurons. First, we note that the FoxP2-cre mouse line is a targeted IRES-cre knock-in behind the endogenous FoxP2 gene, which should give cre expression only in cells that express FoxP2. The faithfulness of this cre line has also been verified in ITCs by others (see Hagihara et al., 2021 Nature, Extended Data Figure

2). Further, we have also performed experiments whereby a cre-depedent reporter (AAV-FLEX-mCherry) was targeted to different ITC clusters and the tissue was immunostained for FoxP2 antibody. The mCherry labeled neurons were entirely within the boundaries of the FoxP2 labeling, and close examination indicates that the vast majority of mCherry+ cells were also FoxP2+ for the three ITC clusters examined in this paper. However, we did observe a minority of cells that expressed mCherry but no observable FoxP2, which could result from failure of detection (FoxP2 is nuclear and mCherry cytoplasmic and can reside on different optical planes) or from non-specific labeling. Nonetheless, the reporter expression appears to largely colocalize with endogenous FoxP2 expression. We have provided images showing colocalization with FoxP2 immunoreactivity in Figure 1b,c and Supplementary figure and included description in the results, see lines 155-167.

2) The authors refer to the mITC as a cluster based on foxp2 expression. This seems quite anatomically distinct from the other groups, almost as if it is simply part of the basalmedial amygdala vs. intercalated cells. This is discussed in the manuscript, but I am not sure simply using the marker of foxp2 is enough to keep calling them mITC. I do note, this is my opinion, and the authors discussion is sufficient, I just wanted to note.

Response: The mITC is indeed somewhat anatomically distinct from the other ITC clusters. The literature on the mITC nucleus is consistent with this, and our anatomical identification of it began with confirming FoxP2 expression in these cells and observing complete similarity with previous delineations of this nucleus. Close examination of the histology in which we stained for FoxP2 and defined the mITC nucleus also shows that although distinct anatomically, being more rostral and ventral to the BLA (thus not within the intermediate and external capsules), it is in fact contiguous with the medial and lateral clusters. We have added an additional comment in the manuscript see line 146150.

ROI quantification:

3) The authors suggest the ‘BNST’ as a key output of several of these structures. There needs to be more precision here. For example in figure 2, the top left panel, there is clear outputs to the BSTLP. but it is not marked or delineated. Whereas in the lower panel, the BSTIA is delineated as BNST. More precise subregion descriptors is required.

Response: We acknowledge that the Bed Nucleus of the Stria Terminalis is composed of multiple subregions over a large area that were not distinguished in our analysis. Given how large this is as a region, with several subnuclei contained within it, along with its fairly diminutive receipt of projections we prefer to focus on the broad areas receiving ITC input and do not distinguish the subregions. However, the extent to which these subregions do or do not receive ITC innervation is observable from the images, as evidenced by the reviewer’s comment. We have clarified this in the text (see line 185188, 210-211) and edited the landmark identification in Figure 2.

4) In this vein, the ROI used for quantification seems a bit imprecise based on the figures. It may be better to align images based on the actual atlas. In addition, the use of the contralateral hemisphere as background would only be appropriate if there was no projection, is this the case?

Response: The ROIs overlaid in the figure images were meant to identify general landmarks, however these were not the ROIs used in quantification. For quantification more precise boundaries were observed in agreement with the Franklin and Paxinos atlas. Aligning microscopy images with atlas templates is a challenging task that is imprecise in our hands (even with some of the modern software packages), which is why we preferred to do it manually. Lastly, we do not observe any contralateral projections. We have clarified these points in the manuscript (see lines 126-129, 185188).

Rabies tracing:

5) There is no control for the rabies tracing. The authors do not quantify starter cells or normalize input strength to starter cells. There is known leakiness in rabies tracing and there is a need for control experiments injecting either viral construct separately and quantifying upward inputs. As is, there is no ability to assess whether the inputs are indeed faithful to the inferred mechanism of action of this approach.

Response: We acknowledge that the number of labeled presynaptic neurons is likely to scale with the number of starter cells, which would be different between ITC clusters, but this does not affect conclusions from within each cluster. We prefer not to normalize the data, but have included a statement to acknowledge this point (see line 192-193).

As for the rabies “leakiness,” The original in vivo report with these viral vectors (Wall et al., 2010 PNAS) performed the control experiment the reviewer is asking for, whereby they injected the helper virus and rabies in cre-negative mice and found “Rabies virus infection was almost completely undetectable in the absence of Cre.” For this reason, we did not include such data in the manuscript. However, we had done some experiments in which the helper virus was not injected and, in these experiments, we did not observe any rabies expression anywhere in the brain. This indicates that the EnvA-DG-rabies does not infect starter cells without the expression of TVA. Further, injection of the helper virus in cre-negative mice prevents GFP expression. Thus, we find no evidence of leaky expression. We have added a statement in the text (see line 180-183) to clarify this issue.
