# Supplementary figures and images for: Anatomical Connectivity of the Intercalated Cells of the Amygdala
Source: eNeuro. 2023 Oct 12;10(10):ENEURO.0238-23.2023. doi: 10.1523/ENEURO.0238-23.2023 (PMC10576262; doi:10.1523/ENEURO.0238-23.2023)

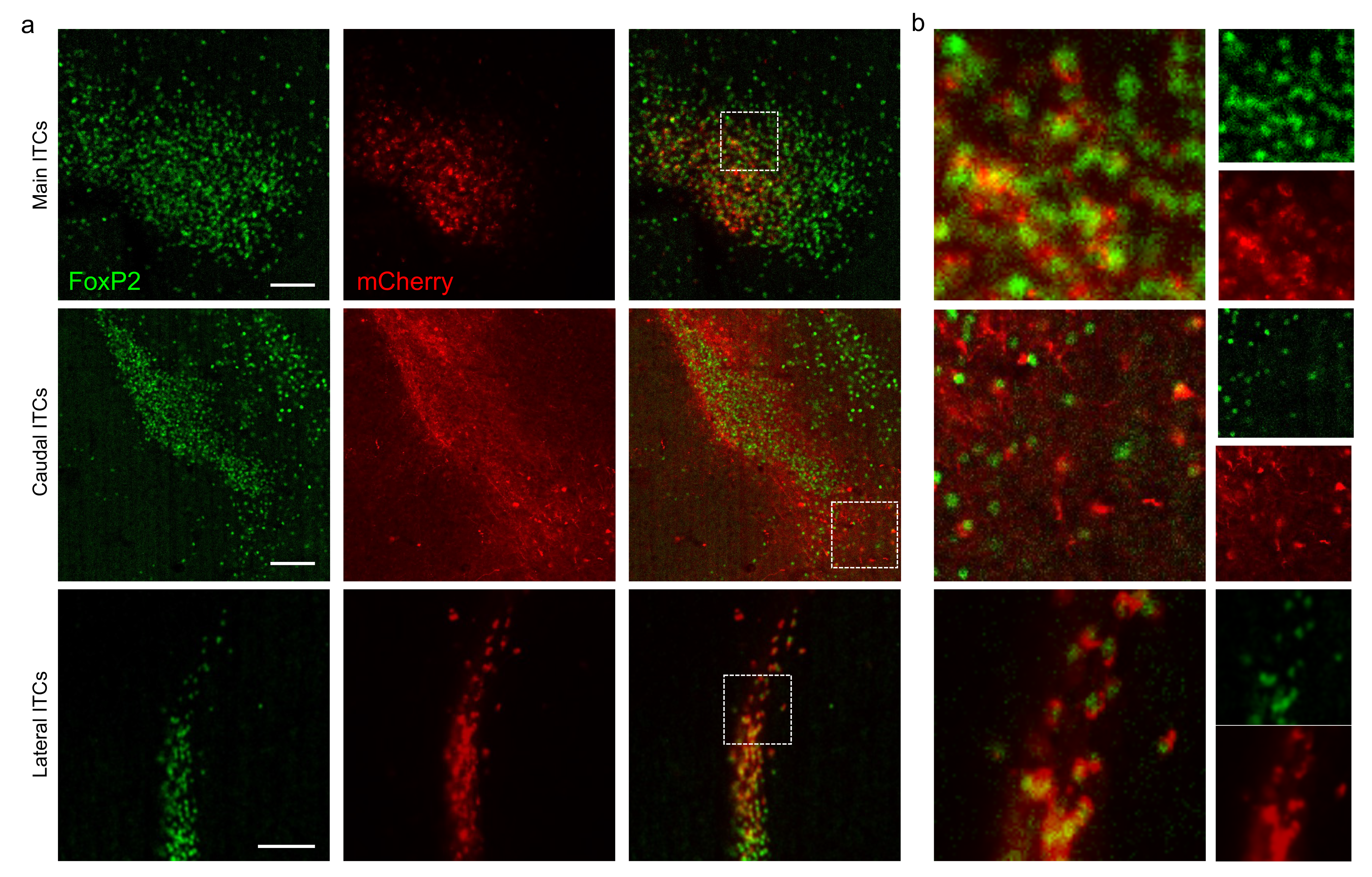

Supplement: Extended Data Figure 1-1 — FoxP2-cre line labels FoxP2 ITCs. A cre-dependent reporter (AAV-FLEX-mCherry) was targeted to different ITC clusters in FoxP2-cre mice. The brain tissue was stained with a FoxP2 antibody to analyze the overlap in reporter and FoxP2 expression. a, Individual channels and merged images for each of the three ITC clusters. Scale bar is 100 μm. b, Zoomed in image from the white boxes in a showing the merged image (left) and individual channel (right). Download Figure 1-1, TIF file. [file enu-eN-NWR-0238-23-s03.tif]
